# Opioid-Associated Postoperative Nausea and Vomiting in Women Undergoing Laparoscopic Hysterectomy: A Network Meta-Analysis

**DOI:** 10.3390/medicina61101728

**Published:** 2025-09-23

**Authors:** Sueyoung Cho, Heesoo Bang, Sangyoon Shin, Hyunjoo Kim, Seohyeon Park, Paul S. Lee, Eunkyung Euni Lee

**Affiliations:** 1 Research Institute of Pharmaceutical Sciences, Natural Products Research Institute, College of Pharmacy, Seoul National University, Seoul 08826, Republic of Korea; sueandi@snu.ac.kr (S.C.); hban2031@snu.ac.kr (H.B.); sys930909@gmail.com (S.S.); 2 College of Pharmacy, Jeju National University, Jeju 63241, Republic of Korea; mary_kim1@snu.ac.kr; 3 Faculty of Pharmaceutical Sciences, University of British Columbia, Vancouver, BC V6T 1Z4, Canada; saigeseohyeonpark@gmail.com; 4 Department of Anesthesiology, University of Southern California, Los Angeles, CA 90033, USA

**Keywords:** laparoscopic hysterectomy, network meta-analysis, opioid, opioid-associated postoperative nausea and vomiting (O-PONV), postoperative pain management

## Abstract

*Background and Objectives*: This systematic review and network meta-analysis evaluated the effects of postoperative opioid use on nausea and vomiting in women undergoing laparoscopic hysterectomy. *Materials and Methods*: A systematic search of PubMed, EMBASE, the Cochrane Library, and RISS was conducted to identify randomized controlled trials that met the eligibility criteria. The Cochrane Risk of Bias 2 tool was used to assess the quality of the included studies. A frequentist network meta-analysis was performed to compare the risks of opioid-associated postoperative nausea and vomiting (O-PONV). Quantitative statistics were presented in forest plots, and the ranking of treatments was determined using the P-score. *Results*: Seventeen studies involving 1315 participants and 18 postoperative analgesic interventions were included. No significant differences were found among the opioid monotherapies—buprenorphine, butorphanol, fentanyl, oxycodone, sufentanil, and tapentadol. However, among the combination therapies, oxycodone/ketorolac therapy was associated with a significantly higher risk of O-PONV than other ketorolac-containing regimens, including dexmedetomidine, remifentanil, and fentanyl. *Conclusions*: No significant differences in O-PONV risk were observed among the six opioid monotherapy groups. An opioid-sparing regimen, such as dexmedetomidine/ketorolac, showed a lower risk of O-PONV than an oxycodone-based regimen, underscoring the importance of incorporating patient-centered considerations, such as patient preference and route of administration, into postoperative pain management.

## 1. Introduction

Hysterectomy is one of the most frequently performed gynecological procedures worldwide, with indications ranging from benign conditions such as uterine fibroids and endometriosis to more serious pathologies, including gynecological cancer and pelvic support disorders [1,2,3]. The choice of surgical approach varies, with abdominal, vaginal, and laparoscopic hysterectomy being the most common techniques [4]. Over the past few decades, laparoscopic hysterectomy has gained widespread adoption as a minimally invasive alternative, largely because of its favorable perioperative outcomes [5,6].

Pain following hysterectomy, as with any surgery, is a well-documented clinical concern, with the intensity ranging from moderate to severe depending on the individual patient [7]. Evidence indicates that laparoscopic hysterectomy is associated with significantly reduced postoperative pain and morbidity compared to abdominal hysterectomy [8,9,10]. As modern perioperative medicine increasingly emphasizes patient-centered care, the optimization of postoperative pain control remains a critical goal for healthcare providers [11]. Effective pain management not only enhances patient comfort but also positively influences quality of life, accelerates functional recovery, and reduces the risk of complications [11,12]. Moreover, minimizing opioid-related adverse events, such as postoperative nausea and vomiting (PONV), is particularly important given its impact on patient satisfaction, recovery trajectory, and healthcare utilization [11].

Opioids remain the cornerstone of perioperative analgesia because of their proven efficacy in managing moderate-to-severe postoperative pain [13]. They are frequently administered either as monotherapy or in combination with non-opioid analgesics to achieve multimodal pain control [14]. However, opioid use is often complicated by adverse effects, most notably opioid-induced nausea and vomiting (OINV) [14]. In addition, evaluating independent effects of OINV after surgery is even more challenging as the incidence and severity of the adverse outcomes can vary considerably among individuals, complicating the ability to make clinically meaningful comparisons between different opioid agents [14]. Notably, women have a higher incidence of PONV than men, making this an especially relevant concern in patients undergoing gynecological surgery, such as laparoscopic hysterectomy [13,15].

Given these considerations, there is a critical need for high-quality evidence to systematically evaluate and quantify the comparative risk of O-PONV among various opioids in this patient population. Such evidence could inform more individualized analgesic strategies, enabling clinicians to balance effective pain relief with the minimization of side effects. Therefore, we conducted a systematic review and network meta-analysis to compare the incidence of nausea or vomiting associated with different postoperative opioid regimens in women who underwent laparoscopic hysterectomy.

## 2. Materials and Methods

This study was conducted according to the Preferred Reporting Items for Systematic Reviews and Meta-Analyses (PRISMA) guidelines [16].

### 2.1. Eligibility

A systematic search of randomized controlled trials (RCTs) was performed based on the following PICO framework:

(1) Population (P): women who underwent laparoscopic hysterectomy,
(2) Intervention/Comparison (I/C): any pairwise comparisons of opioids or comparisons of opioids with non-opioid analgesics for postoperative pain management, and
(3) Outcome (O): postoperative nausea or vomiting.

### 2.2. Search Strategy

We searched PubMed (NLM^®^), EMBASE (OvidSP), the Cochrane Library (Cochrane), and RISS (Korea Education and Research Information Service) from their respective inception dates to July 2025. The complete search strategy is presented in Table S1. To ensure comprehensiveness, all available opioid agents listed in accredited pharmaceutical databases, including Micromedex (Merative™, Ann Arbor, MI, USA), were included as keywords across all search fields. All retrieved studies were managed using EndNote 20 (Clarivate, Philadelphia, PA, USA) to facilitate systematic screening and identification. A language filter was applied only to English-language publications.

### 2.3. Study Selection

Two authors (S.C. and H.B.) independently screened and reviewed the retrieved articles to assess their eligibility based on the inclusion criteria predefined in the PICO statement. Disagreements were resolved through discussion with a third author (E.L.) until a consensus was reached. Studies were excluded in the following order: (1) non-RCTs; (2) no postoperative opioid use in any study arm; (3) non-pharmacological interventions (e.g., diet, exercise) or procedures (e.g., transversus abdominis plane block) in any study arm; and (4) studies comparing the same opioid by route, dose, or timing of administration.

The included studies were summarized by the country of origin, number of participants, drugs used for postoperative pain management, and reported incidence of nausea or vomiting.

### 2.4. Definition of Independent and Dependent Variables

The independent variable in this study was the use of postoperative opioid or non-opioid analgesics, administered either as monotherapy or in combination. An intervention was considered postoperative if it was administered at the end of the surgery, in the post-anesthesia care unit (PACU), or was explicitly described. The dependent variable was the incidence of nausea or vomiting reported at any time within 72 h after surgery.

For studies that reported nausea and vomiting as separate outcomes, higher value was used for subsequent reporting and analysis as we considered the adverse effects of either nausea or vomiting negatively affected patients’ quality of life. When the incidence data were reported at multiple time points, the maximum value was selected as we considered the highest potential of causing adverse outcome was clinically important. If the incidence was presented using severity scores, a score of 0 was categorized as the absence of nausea or vomiting, whereas scores above zero were categorized as the presence of nausea or vomiting.

### 2.5. Risk-of-Bias Assessment

The risk of bias in the included studies was assessed using the Cochrane Risk of Bias 2 tool [17]. This tool includes five domains: bias arising from the randomization process, deviations from intended interventions, missing outcome data, measurement of the outcome, and selection of the reported result. Each domain was rated as “low,” “some concerns,” or “high” for each study. Two authors (H.aB. and S.C.) independently assessed the risk of bias in all included studies in accordance with the guidelines. Any discrepancies were resolved through discussions with a third author (E.L.).

### 2.6. Statistical Analysis

A frequentist network meta-analysis (NMA) was conducted using the “netmeta” package in R version 4.4.2 (R Foundation for Statistical Computing, Vienna, Austria) and the “network” package in STATA/SE 19 (StataCorp, College Station, TX, USA) [18,19]. Study characteristics, including design, interventions, sample size, and outcome measures, were extracted and summarized. Heterogeneity among the studies was evaluated using the I^2^ statistic, and all analyses were performed using a random-effects model. Inconsistency was tested using a design-by-treatment interaction model. Quantitative results are presented in league tables and forest plots. The ranking of treatments was determined using the P-score. If incidence data were reported only in graphical form in the source studies, numerical data were extracted using WebPlotDigitizer (version 5.0; Automeris, TX, USA). Potential publication bias was evaluated using comparison-adjusted funnel plots to graphically assess asymmetry.

## 3. Results

### 3.1. Selected Studies

**Figure 1:**
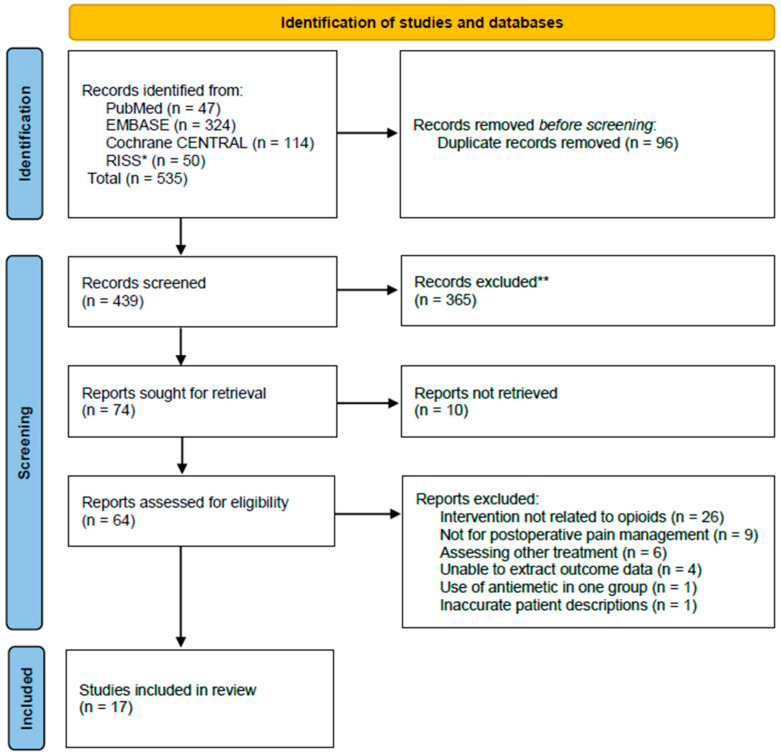
Study flow of study screening and eligibility. * RISS: Research Information Sharing Service database operated by the Korea Education & Research Information Service. ** Excluded during title/abstract screening: not randomized controlled trial, non-human study, non-English, non-drug comparison study (e.g., diet, exercise).

A total of 535 relevant studies were identified in the initial search, of which 96 duplicate studies were excluded. After screening by title and abstract, 365 studies were excluded. An additional ten studies were excluded because their full texts were unavailable. Following full-text assessment of the remaining studies, 17 were deemed eligible for quantitative analysis (Figure 1).

### 3.2. Study Characteristics

The 17 included studies comprised 1315 participants. Nine studies were conducted in Republic of Korea [20,21,22,23,24,25,26,27,28], four in China [29,30,31,32], two in Norway [33,34], one in Italy [35], and one in India [36]. Oxycodone was the most commonly used analgesic (Table 1).

### 3.3. Risk of Bias Assessment

**Figure 2:**
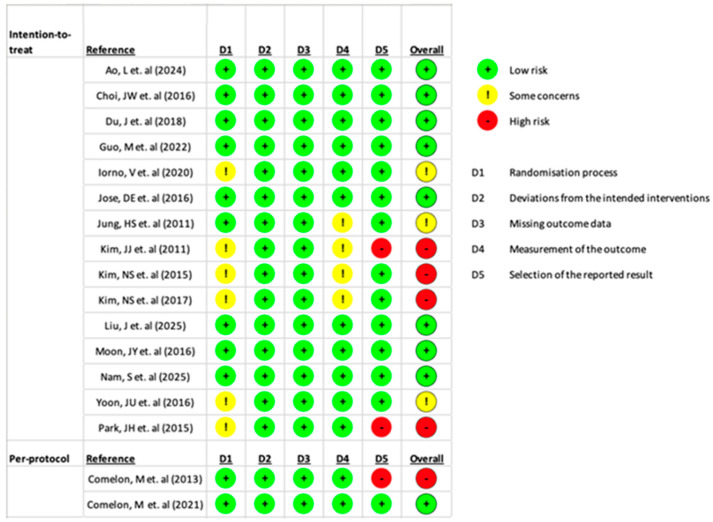
Summary of risk of bias assessment [20,21,22,23,24,25,26,27,28,29,30,31,32,33,34,35,36].

In total, five studies (29%) had a high risk of bias, and three studies (18%) were rated as having some concerns. Of the six studies included in the NMA of monotherapy [20,22,31,32,33,36], one was judged to have a high risk of bias [22], one was rated as having some concerns [20], and the remaining studies were assessed as having a low risk of bias. Of the four studies included in the NMA of combination regimens [21,23,24,27], three were determined to have a high risk of bias [21,23,27] (Figure 2).

### 3.4. Network Meta Analyses (NMA)

We conducted an NMA of 17 studies, representing 18 different postoperative analgesic regimens. Most of the comparisons were connected to either oxycodone monotherapy or fentanyl/ketorolac combination therapy (Figure S1). The risk associated with dexmedetomidine/ketorolac combination therapy was significantly lower than that associated with dexmedetomidine monotherapy (OR = 0.0105 [95% CI: 0.0001–0.7864], I^2^ = 0%, Figure S1). However, the total administered doses of dexmedetomidine could not be compared because one study reported only a range of infusion rates.

**Figure 3:**
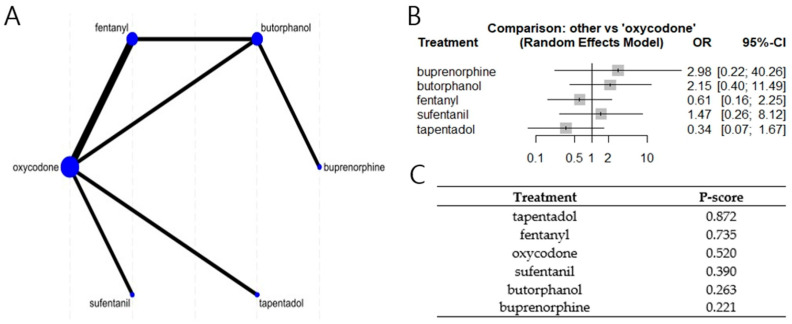
Network meta-analysis of five studies including opioid monotherapies (**A**). Network geometry; (**B**). Forest plot comparing the risk of nausea or vomiting; (**C**). P-score ranking.

Of the six studies comparing postoperative opioid monotherapy pairs, five were included in the monotherapy analysis. These studies represented six different postoperative analgesic regimens. The NMA findings showed no significant differences in the risk of nausea or vomiting (I^2^ = 41.6%; Figure 3). There was no evidence of inconsistency (*p* = 0.19).

**Figure 4:**
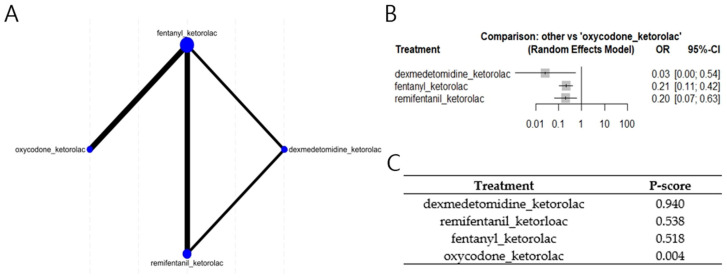
Network meta-analysis of four studies including combination regimens with ketorolac (**A**). Network geometry; (**B**). Forest plot comparing the risk of nausea or vomiting; (**C**). P-score Ranking.

Four studies of ketorolac-containing combination regimens were available for NMA synthesis. Compared with the oxycodone/ketorolac combination regimen, other ketorolac-containing combination regimens showed a lower risk of nausea or vomiting: dexmedetomidine/ketorolac (odd ratio [OR] = 0.0267 [95% confidence interval (CI): 0.0013–0.5442]), fentanyl/ketorolac (OR = 0.2106 [95% CI: 0.1059–0.4188]), and remifentanil/ketorolac (OR = 0.2034 [95% CI: 0.0661–0.6259]) (I^2^ = 0%; Figure 4). This inconsistency was not statistically significant (*p* = 0.96).

Detailed pairwise comparisons of monotherapies, ketorolac-containing combination regimens, and the overall network are provided in league tables (Figure S2). Three funnel plots were generated to evaluate potential publication bias (Figure S3). The plots for monotherapies and ketorolac-containing combination regimens showed asymmetry, indicating a possible risk of publication bias.

## 4. Discussion

This systematic review and network meta-analysis, which included 17 trials and 1315 participants, examined the incidence of O-PONV using opioid monotherapy and combination regimens containing non-opioid analgesics. Eighteen active treatment regimens for postoperative pain management after laparoscopic hysterectomy were assessed. Among the 17 studies, 9 were assessed as having a low risk of bias, 3 as having some concerns, and 5 as having a high risk. This distribution allowed for a reasonably robust synthesis of evidence through NMA, thereby enhancing confidence in the comparative estimates of O-PONV risk.

One notable finding was the absence of significant differences in O-PONV risk among the six opioid monotherapies studied. Oxycodone, a commonly used opioid, did not differ significantly from buprenorphine, butorphanol, fentanyl, sufentanil, or tapentadol in terms of associated nausea or vomiting. This aligns with previous research reporting that the choice of opioid had little effect on O-PONV risk, except for buprenorphine, which was the only opioid shown to have a higher risk than intravenous (IV) patient-controlled analgesia with morphine [13]. However, in our analysis, buprenorphine was administered epidurally, a route associated with a lower O-PONV risk than IV administration [37,38], which may explain this discrepancy.

In contrast to monotherapy, certain ketorolac-based combination regimens have demonstrated more favorable O-PONV profiles. Dexmedetomidine/ketorolac, fentanyl/ketorolac, and remifentanil/ketorolac were associated with significantly lower O-PONV risk compared with oxycodone/ketorolac. Notably, dexmedetomidine/ketorolac demonstrated the most pronounced risk reduction, likely reflecting their non-opioid mechanisms of action [20]. As three of the four combination regimens were at high risk of bias, the finding should require careful interpretation and further studies with high validity are needed.

Our findings underscore the importance of multimodal analgesic strategies, particularly those that minimize opioid exposure while maintaining effective pain control. The results suggest that the choice of analgesic regimen should not rely solely on opioid type or potency but should prioritize patient-centered care, factoring in patient preferences, prior O-PONV history, and optimal routes of administration, to optimize both pain control and patient comfort in the postoperative setting. Tailoring analgesic strategies to opioid-sparing and multimodal approaches may reduce postoperative complications and improve patient recovery. These findings may serve as a reference for clinicians in various countries when selecting opioid and non-opioid analgesics for patients at a higher risk of developing O-PONV.

A key strength of this study was its originality in addressing a clinically important and underexplored research question: the comparative impact of various analgesic strategies on O-PONV, specifically in the context of laparoscopic hysterectomy. Although previous meta-syntheses have largely focused on the efficacy of opioids for pain control across different surgical procedures [8,12,39,40], few studies have specifically examined O-PONV in this patient population.

Although our search identified various opioid monotherapies and combination regimens, the use of NMA allowed us to overcome the limitations of limited direct head-to-head comparisons. The NMA enabled indirect comparisons across regimens and facilitated the quantitative synthesis of the available evidence. By incorporating both direct and indirect comparisons, the NMA provided a more comprehensive evaluation and generated clinical evidence regarding the relative effects of opioid regimens on O-PONV. Using this approach, the relative effectiveness of different opioid regimens was estimated.

Our study had several limitations. First, the analysis did not include a sufficient number of studies to evaluate the dose-dependent effects of opioids, variations in routes of administration, the timing of outcome assessments, or baseline patient characteristics. Because opioid analgesics are known to exert dose-dependent effects on nausea and vomiting, future studies should investigate these relationships more explicitly [41]. Second, the moderate level of heterogeneity observed from the analysis including studies with monotherapies may be attributable to variations in opioid regimens and other treatment-related factors. Furthermore, the varying granularity of dosage information across studies, and potential lingering effects of perioperative analgesia warrant cautious interpretation of the results. Third, the included studies indicated that opioid prescription practices after laparoscopic hysterectomy varied geographically. For example, in a previous study, oxycodone was the most frequently used opioid in high-income countries, whereas tramadol and codeine were more commonly used in lower-resource settings [42]. Differences in available medications, clinician preferences, and postoperative pain management strategies may have influenced the observed outcomes. As most analyzed studies were from East Asia, additional studies across other regions, such as North America and Europe, and beyond are required to broaden applicability. Lastly, recognizing the multifactorial etiology of PONV—and opioid use as one of its contributing factors—distinguishing OINV from PONV or other treatment-related adverse effects can be challenging [7]. Our study defined the study outcome as opioid-associated postoperative nausea and vomiting (O-PONV) rather than OINV to more accurately represent nausea and vomiting events linked to postoperative opioid use while considering other factors that were potentially associated with nausea and vomiting such as patient characteristics and surgery settings. Future research should aim to collect more standardized and detailed data on opioid type, dose, and route of administration, as well as employ uniform definitions of OINV, to enable more robust comparisons and guide optimized postoperative pain management. Furthermore, additional studies using real-world data to capture patient preferences and humanistic outcomes are warranted to better inform patient-centered postoperative pain management strategies.

## 5. Conclusions

We found no significant differences in the risk of nausea or vomiting among the six opioid monotherapies, underscoring the importance of incorporating patient-centered considerations, such as the preferred agent and route of administration, into postoperative pain management. An opioid-sparing regimen of dexmedetomidine combined with ketorolac may suggest a lower risk of O-PONV than an oxycodone-based regimen. This finding highlights the potential advantages of multimodal approach with non-opioid analgesia and underscores the need for further studies.

## Figures and Tables

**Table 1:** Characteristics of included studies.

Study	Country	Arms	N	Treatment	Outcome
Ao, L., 2024 [29]	China	DK	34	Dexmedetomidine 2 μg/kg and ketorolac 3 mg/kg with NS IV PCA: 0.5 mL bolus, 2 mL/h basal, 15 min lockout	48 h: PONV 2/34
		SUF	32	Sufentanil 1.5 μg/kg with NS IV PCA: 0.5 mL bolus, 2 mL/h basal, 15 min lockout	48 h: PONV 8/32
Choi, J.W., 2016 [24]	Republic of Korea	FK	30	Fentanyl: LD 1.0 μg/kg, continuous infusion 0.4 μg/kg/h Ketorolac: IV single dose 30 mg	30 min: N 3 (10%), V 3 (10%)
		RK	30	Remifentanil: LD 1.0 μg/kg, continuous infusion 0.08 μg/kg/h Ketorolac: IV single dose 30 mg	30 min: N 3 (10%), V 2 (6.7%)
		DK	30	Dexmedetomidine: LD 1 μg/kg, continuous infusion 0.5 μg/kg/h Ketorolac: IV single dose 30 mg	30 min: N 0 (0%), V 0 (0%)
Comelon, M., 2013 [34]	Norway	ON	40	Oxycodone/naloxone PR 10 mg/5 mg every 12 h for a total of 3 days	0–4 h: N 7%, V 0% 4–24 h: N 12%, V 3%
		O	45	Oxycodone PR 10 mg every 12 h for a total of 3 days	0–4 h: N 7%, V 0% 4–24 h: N 13%, V 3%
Comelon, M., 2021 [33]	Norway	T	37	Oral extended-release tapentadol 50 mg	1 h: N 16.2%, V 2.7% 2 h: N 10.8%, V 0% 3 h: N 8.1%, V 0% 24 h: N 21.6%, V 18.9%
		O	36	Oral extended-release oxycodone 10 mg	1 h: N 8.3%, V 0% 2 h: N 8.3%, V 5.6% 3 h: N 19.4%, V 5.6% 24 h: N 44.4%, V 27.8%
Du, J., 2018 [30]	China	CON	40	Butorphanol 10 mg PCIA: 0.5 mL bolus, 2 mL/h basal, 15 min lockout	24 h: N 12 (30.0%), V 7 (17.1%)
		DEX	41	Butorphanol 10 mg, dexmedetomidine 300 μg PCIA: 0.5 mL bolus, 2 mL/h basal, 15 min lockout	24 h: N 7 (17.1%), V 2 (4.9%)
Guo, M., 2022 [31]	China	F	39	Fentanyl 8.3 μg/kg IV PCA: 3 mL bolus, 2 mL/h basal, 15 min lockout	48 h: N 4 (10.3%), V 0 (0%)
		O	36	Oxycodone 0.5 mg/kg IV PCA: 3 mL bolus, 2 mL/h basal, 15 min lockout	48 h: N 8 (21.6%), V 2 (5.4%)
		B	37	Butorphanol 0.16 mg/kg IV PCA: 3 mL bolus, 2 mL/h basal, 15 min lockout	48 h: N 3 (8.3%), V 0 (0%)
Iorno, V., 2020 [35]	Italy	OXN	42	PR oxycodone/naloxone (10 mg/5 mg), every 12 h up to 48 h postoperatively	Day0: N 7, V 2 Day1: N 3, V 0 Day2: N 1, V 0 Day3: N 1, V 0 *
		M	41	Morphine 0.2~0.4 mg/kg/day continuous infusion 48 h postoperatively Ketorolac 30 mg IV three times a day	Day0: N 10, V 4 Day1: N 7, V 2 Day2: N 3, V 0 Day3: N 0, V 0 *
Jose, D.E., 2016 [36]	India	A	30	Buprenorphine 0.3 mg with 10 mL NS injected via epidural catheter	12 h: NV 4 (13%)
		B	30	Butorphanol 1 mg with 10 mL NS injected via epidural catheter	12 h: NV 3 (10%)
Jung, H.S., 2011 [20]	Republic of Korea	D	25	Dexmedetomidine: LD 1 μg/kg, continuous infusion 0.2~0.7 μg/kg/h	30 min: N 3 (13%), V 2 (8%)
		R	25	Remifentanil: LD 0.8~1.2 μg/kg, continuous infusion 0.05~0.1 μg/kg/h	30 min: N 0 (0%), V 0 (0%)
Kim, J.J., 2011 [21]	Republic of Korea	R	20	Remifentanil IV PCA: LD 1 μg/kg, 0.375 μg/kg bolus, 0.025 μg/kg/min basal	Score 0 (none): 8 (40%) Score 1 (mild): 7 (35.0%) Score 2 (moderate): 4 (20.0%) Score 3 (severe): 0 (0%) Score 4 (vomiting): 1 (5%)
		RK1	19	Remifentanil IV PCA: LD 0.6 μg/kg, 0.225 μg/kg bolus, 0.015 μg/kg/min basal Ketorolac: LD 30 mg, 0.01 μg/kg bolus, 0.04 μg/kg/h basal, 15 min lockout	Score 0 (none): 12 (63.2%) Score 1 (mild): 4 (21.1%) Score 2 (moderate): 2 (10.5%) Score 3 (severe): 0 (0%) Score 4 (vomiting): 1 (5.3%)
		RK2	20	Remifentanil: LD 0.3 μg/kg, 0.1125 μg/kg bolus, 0.0075 μg/kg/min basal Ketorolac: LD 30 mg, 0.01 μg/kg bolus, 0.04 μg/kg/h basal, 15 min lockout	Score 0 (none): 8 (40.0%) Score 1 (mild): 4(20.0%) Score 2 (moderate): 5 (25.0%) Score 3 (severe): 3 (15.0%) Score 4 (vomiting): 0 (0%)
		F	20	Fentanyl: LD 1 μg/kg, 0.075 μg/kg bolus, 0.3 μg/kg/h basal, 15 min lockout Ketorolac: LD 30 mg, 0.01 μg/kg bolus, 0.04 μg/kg/h basal, 15 min lockout	Score 0 (none): 10 (50.0%) Score 1 (mild): 4 (20.0%) Score 2 (moderate): 4 (20.0%) Score 3 (severe): 1 (5.0%) Score 4 (vomiting): 1 (5.0%)
Kim. N.S., 2015 [23]	Republic of Korea	F	30	Fentanyl 700 μg (LD 100 μg) Ketorolac 150 mg (LD S30 mg) IV PCA: 0.5 mL bolus, 14 μg/h basal, 15 min lockout	0.5 h: N 1 (3.3%), V 0 (0%) 2 h: N 3 (10%), V 1 (3.3%) 4 h: N 6 (20%), V 1 (3.3%) 8 h: N 6 (20%), V 0 (0%) 24 h: N 4 (13.3%), V 2 (6.7%) 48 h: N 4 (13.3%), V 1 (3.3%)
		O	30	Oxycodone 70 mg (LD 10 mg) Ketorolac 150 mg (LD 30 mg) IV PCA: 0.5 mL bolus, 1.4 mg/h basal, 15 min lockout	0.5 h: N 1 (3.3%), V 0 (0%) 2 h: N 4 (13.3%), V 0 (0%) 4 h: N 14 (46.7%), V 1 (3.3%) 8 h: N 13 (43.3%), V 2 (6.7%) 24 h: N 12 (40.0%), V 4 (13.3%) 48 h: N 12 (40.0%), V 3 (10.0%)
Kim, N.S., 2017 [27]	Republic of Korea	F	63	Fentanyl 700 μg (LD 100 μg) Ketorolac 150 mg (LD 30 mg) IV PCA: 0.5 mL bolus, 14 μg/h basal, 15 min lockout	0.5 h: N 2 (3.2%, V 0 (0%) 4 h: N 8 (12.7%), V 3 (4.8%) 8 h: N 9 (14.3%), V 1 (1.6%) 24 h: N 9 (14.3%), V 5 (7.9%) 48 h: N 2 (3.2%), V 3 (4.8%)
		O	64	Oxycodone 52.5 mg (LD 7.5 mg) Ketorolac 150 mg (LD 30 mg) IV PCA: 0.5 mL bolus, 1050 μg/h basal, 15 min lockout	0.5 h: N 2 (3.1%), V 0 (0%) 4 h: N 21 (32.8%), V 2 (3.1%) 8 h: N 21 (32.8%), V 9 (14.1%) 24 h: N 31 (48.4%), V 8 (12.5%) 48 h: N 18 (28.1%), V 2 (3.1%)
Liu, J., 2025 [32]	China	O	28	Oxycodone 0.5 mg/mL IV PCA: 4 mL bolus, 1 mL/h basal, 15 min lockout	48 h: N 6/28 (21.4%), V 3/28 (10.7%)
		C	28	Sufentanil 0.5 μg/mL IV PCA: 4 mL bolus, 1 mL/h basal, 15 min lockout	48 h: N 8/28 (28.6%), V 7/28 (25.0%)
Moon, J.Y., 2016 [25]	Republic of Korea	A	27	Fentanyl 1000 μg (LD 20 μg) IV PCA: 1 mL bolus, 5 min lockout, 10 mL/h max, total daily max 60 mL (no basal infusion)	48 h: NV 17 (59.3%)
		B	28	Fentanyl 500 μg (LD 10 μg) Nefopam 200 mg (LD 4 mg) IV PCA: 1 mL bolus, 5 min lockout, 10 mL/h max, total daily max 60 mL (no basal infusion)	48 h: NV 18 (64.3%)
		C	26	Fentanyl 500 μg (LD 10 μg) Nefopam 400 mg (LD 8 mg) IV PCA: 1 mL bolus, 5 min lockout, 10 mL/h max, total daily max 60 mL (no basal infusion)	48 h: NV 18 (69.2%)
Nam, S., 2025 [28]	Republic of Korea	C	42	Fentanyl 500 μg + nefopam 80 mg IV PCA IV PCA: 1 mL bolus, 0.5 mL/h basal, 10 min lockout	24 h: N 15 (35.7%), V 1 (2.4%)
		T	41	Acetaminophen IV 1 g Fentanyl 500 μg + nefopam 80 mg IV PCA IV PCA: 1 mL bolus, 0.5 mL/h basal, 10 min lockout	24 h: N 15 (26.6%), V 4 (9.8%)
Park, J.H., 2015 [22]	Republic of Korea	O	37	Oxycodone IV PCA: 0.9 mg bolus, 0.9 mg/h basal, 15 min lockout	48 h: N 11, V 3
		F	32	Fentanyl IV PCA: 15 μg bolus, 15 μg/h basal, 15 min lockout	48 h: N 4, V 1
Yoon, J.U., 2016 [26]	Republic of Korea	A	30	Morphine 60 mg + ketorolac 180 mg IV PCA: 1 mL bolus, 1 mL/h basal, 15 min lockout	PACU: N 7 (23.3%), V 0 (0%) 12 h: N 14 (46.7%), V 3 (10.0%) 24 h: N 12 (40.0%), V 2 (6.7%) 48 h: N 9 (30.0%), V 0 (0%)
		B	30	Nefopam 200 mg IV PCA: 1 mL bolus, 1 mL/h basal, 15 min lockout	PACU: N 6 (20.0%), V 0 (0%) 12 h: N 3 (10.0%), V 1 (3.3%) 24 h: N 3 (10.0%), V 0 (0%) 48 h: N 3 (10.0%), V 0 (0%)

IV: intravenous; LD: loading dose; N: nausea; NS: normal saline; PONV: postoperative nausea and vomiting; PCA: patient-controlled analgesia; PCIA: patient-controlled intravenous analgesia; PR: per rectum; V: vomiting. * Graphic source data converted to numerical value.

## Data Availability

No new data were created or analyzed in this study. Data sharing is not applicable to this article.

## Supplementary Materials

The following supporting information can be downloaded at: https://www.mdpi.com/article/10.3390/medicina61101728/s1, Table S1: Full Search Strategy; Figure S1: Network meta-analysis of all studies; Figure S2: League table based on network odds ratios; Figure S3: Comparison-adjusted funnel plot assessing publication bias.

## Abbreviations

The following abbreviations are used in this manuscript:

O-PONV	Opioid-associated postoperative nausea and vomiting
OINV	Opioid-induced nausea and vomiting
PONV	Postoperative nausea and vomiting
RCT	Randomized controlled trial
PRISMA	Preferred Reporting Items for Systematic Reviews and Meta-Analyses
PICO	Population, Intervention, Comparison, and Outcome
RISS	Research Information Sharing Service
PACU	Post-Anesthesia Care Unit
NMA	Network meta-analysis
STATA/SE	Statistical software for data science/special edition
OR	Odds ratio
CI	Confidence interval
