# Supplementary material for: Opioid-Associated Postoperative Nausea and Vomiting in Women Undergoing Laparoscopic Hysterectomy: A Network Meta-Analysis
Source: Medicina (Kaunas). 2025 Sep 23;61(10):1728. doi: 10.3390/medicina61101728 (PMC12565775; doi:10.3390/medicina61101728)
Supplement: Supplementary file 1 [file medicina-61-01728-s001.zip › medicina-3850253-supplementary.pdf]

medicina

MDPI

# Supplementary Materials

Table S1. Full Search Strategy

| | | |
|---|---|---|
| PubMed (25.07.22) | | |
| #1 | (((((((((((((((((((((Alfentanil) OR (Buprenorphine)) OR (Butorphanol)) OR (Codeine)) OR (Dezocine)) OR (Difelikefalin)) OR (Fentanyl)) OR (Hydrocodone)) OR (Hydromorphone)) OR (Ketobemidone)) OR (Levomethadyl)) OR (Meperidine)) OR (Methadone)) OR (Morphine)) OR (Nalbuphine)) OR (Oxycodone)) OR (Oxymorphone)) OR (Pentazocine)) OR (Propoxyphene)) OR (Remifentanil)) OR (Sufentanil)) OR (Tapentadol)) OR (Tramadol) [All Fields] | 137210 |
| #2 | (Nausea) OR (Vomiting) [All Fields] | 122298 |
| #3 | (Laparoscopic) [All Fields] | 169318 |
| #4 | (Hysterectomy) [All Fields] | 49058 |
| #5 | #1 AND #2 AND #3 AND #4 | 47 |
| **Cochrane Library** (25.07.22) | | |
| #1 | alfentanil or buprenorphine or butorphanol or codeine or dezocine or difelikefalin or fentanyl or hydrocodone or hydromorphone or ketobemidone or levomethadyl or meperidine or methadone or morphine nalbuphine or oxycodone or oxymorphone or pentazocine or propoxyphene or remifentanil or sufentanil or tapentadol or tramadol | 47531 |
| #2 | nausea or vomiting | 70315 |
| #3 | laparoscopic | 27975 |
| #4 | hysterectomy | 9692 |
| #5 | #1 AND #2 AND #3 AND #4 | 114 |
| **EMBase** (25.07.22) | | |
| #1 | 'alfentanil'/exp OR 'alfentanil' OR 'buprenorphine'/exp OR 'buprenorphine' OR 'butorphanol'/exp OR 'butorphanol' OR 'codeine'/exp OR 'codeine' OR 'dezocine'/exp OR 'dezocine' OR 'difelikefalin'/exp OR 'difelikefalin' OR 'fentanyl'/exp OR 'fentanyl' OR 'hydrocodone'/exp OR 'hydrocodone' OR 'hydromorphone'/exp OR 'hydromorphone' OR 'levacetylmethadol'/exp OR 'levacetylmethadol' OR 'pethidine'/exp OR 'pethidine' OR 'methadone'/exp OR 'methadone' OR 'morphine'/exp OR 'morphine' OR 'nalbuphine'/exp OR 'nalbuphine' OR 'oxycodone'/exp OR 'oxycodone' OR 'oxymorphone'/exp OR 'oxymorphone' OR 'pentazocine'/exp OR 'pentazocine' OR 'dextropropoxyphene'/exp OR 'dextropropoxyphene' OR 'remifentanil'/exp OR 'remifentanil' OR 'sufentanil'/exp OR 'sufentanil' OR 'tapentadol'/exp OR 'tapentadol' OR 'tramadol'/exp OR 'tramadol' OR 'ketobemidone'/exp OR 'ketobemidone' | 371602 |
| #2 | 'nausea' OR 'vomiting' | 533437 |
| #3 | 'laparoscopic' | 282119 |
| #4 | 'hysterectomy' | 114427 |
| #5 | #1 AND #2 AND #3 AND #4 | 324 |
| **RISS*** (25.07.22) | | |
| #1 | 전체**: hysterectomy <OR> 자궁절제술** <OR> 자궁적출술** | |
| #2 | 전체: laparoscopic | |

 https://doi.org/10.3390/medicina61101728

| | | |
|---|---|---|
| #3 | 전체: Opioid <OR> 오피오이드** <OR> alfentanil <OR> buprenorphine <OR> butorphanol <OR> codeine <OR> dezocine <OR> difelikefalin <OR> fentanyl <OR> hydrocodone <OR> hydromorphone <OR> ketobemidone <OR> levomethadyl <OR> meperidine <OR> methadone <OR> morphine <OR> nalbuphine <OR> oxycodone <OR> oxymorphone <OR> pentazocine <OR> propoxyphene <OR> remifentanil <OR> sufentanil <OR> tapentadol <OR> tramadol | |
| #4 | #1 AND #2 AND #3 (international: 24, domestic: 26) | **50** |

*_RISS: Research Information Sharing Service_ database operated by the Korea Education & Research Information Service
**Korean translation of 'all fields', 'hysterectomy', 'opioid'

Figure S1. Network meta-analysis of all studies

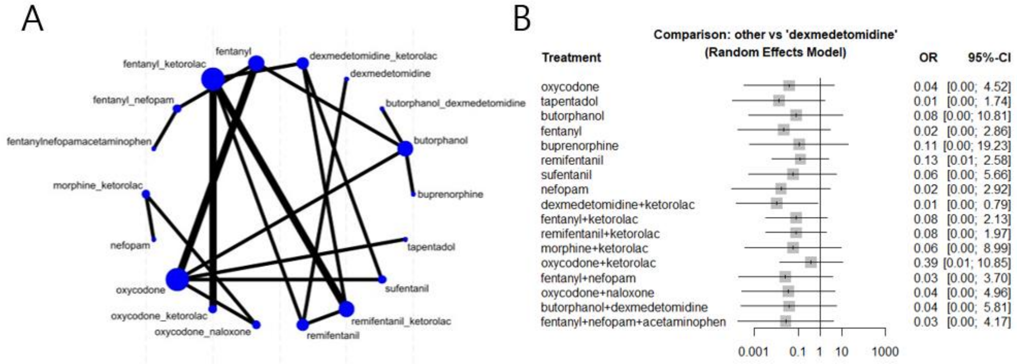


C

| Treatment | P-score |
|---|---|
| dexmedetomidine+ketorolac | 0.854 |
| tapentadol | 0.842 |
| nefopam | 0.762 |
| fentanyl | 0.725 |
| fentanyl+nefopam | 0.657 |
| fentanyl+nefopam+acetaminophen | 0.639 |
| oxycodone+naloxone | 0.562 |
| butorphanol+ketorolac | 0.553 |
| oxycodone | 0.531 |
| remifentanil+ketorolac | 0.441 |
| fentanyl+ketorolac | 0.430 |
| sufentanil | 0.422 |
| morphine+ketorolac | 0.412 |
| remifentanil | 0.332 |
| butorphanol | 0.320 |
| buprenorphine | 0.285 |
| oxycodone+ketorolac | 0.132 |
| dexmdetomidine | 0.102 |

(A) Network geometry.

(B) Forest plot comparing the risk of post-operative OINV between all mono- and combination therapies.

(C) P-score ranking.

Figure S2. League table based on network odds ratios

A

| | | | | | |
|---|---|---|---|---|---|
| tapentadol | | | | | |
| 1.76 (0.23 to 13.67) | fentanyl | | | | |
| 2.90 (0.60 to 14.04) | 0.61 (0.16 to 2.25) | oxycodone | | | |
| 4.25 (0.41 to 43.60) | 0.41 (0.05 to 3.57) | 0.68 (0.12 to 3.78) | sufentanil | | |
| 6.24 (0.62 to 62.28) | 3.54 (0.69 to 18.11) | 2.15 (0.40 to 11.49) | 1.47 (0.13 to 16.09) | butorphanol | |
| 8.63 (0.41 to 181.33) | 4.90 (0.37 to 64.43) | 2.98 (0.22 to 40.26) | 2.03 (0.09 to 45.80) | 1.38 (0.19 to 10.16) | buprenorphine |

B

| | | | |
|---|---|---|---|
| dexmedetomidine+ketorolac | | | |
| 0.13 (0.01 to 2.47) | remifentanil+ketorolac | | |
| 0.13 (0.01 to 2.39) | 1.04 (0.43 to 2.52) | fentanyl+ketorolac | |
| 0.03 (0.00 to 0.54) | 4.92 (1.60 to 15.13) | 0.21 (0.11 to 0.42) | oxycodone+ketorolac |

(A) League table of monotherapies.

(B) League table of combination therapies with ketorolac.

(C) Overall league table of all included comparisons (next page).

C

| | | | | | | | | | | | | | | | | | |
|---|---|---|---|---|---|---|---|---|---|---|---|---|---|---|---|---|---|
| dexmedetomidine +ketorolac | | | | | | | | | | | | | | | | | |
| 0.80<br>[0.08; 7.82] | tapentadol | | | | | | | | | | | | | | | | |
| 0.64<br>[0.04; 11.39] | 1.24<br>[0.13; 11.99] | nefopam | | | | | | | | | | | | | | | |
| 0.48<br>[0.05; 4.63] | 1.66<br>[0.40; 6.86] | 1.34<br>[0.14; 12.71] | fentanyl | | | | | | | | | | | | | | |
| 0.41<br>[0.03; 4.79] | 1.95<br>[0.35; 10.86] | 1.57<br>[0.14; 18.22] | 0.85<br>[0.32; 2.23] | fentanyl+ nefopam | | | | | | | | | | | | | |
| 0.39<br>[0.03; 5.40] | 2.03<br>[0.29; 14.05] | 1.63<br>[0.12; 22.18] | 0.82<br>[0.22; 3.05] | 0.96<br>[0.39; 2.36] | fentanyl+nefopam +acetaminophen | | | | | | | | | | | | |
| 0.30<br>[0.03; 3.28] | 2.69<br>[0.53; 13.76] | 0.46<br>[0.10; 2.23] | 0.62<br>[0.12; 3.08] | 0.73<br>[0.11; 4.73] | 0.75<br>[0.09; 6.02] | oxycodone +naloxone | | | | | | | | | | | |
| 3.57<br>[0.26; 48.55] | 2.84<br>[0.42; 19.44] | 2.29<br>[0.17; 30.79] | 1.71<br>[0.35; 8.39] | 1.46<br>[0.23; 9.34] | 1.40<br>[0.18; 11.03] | 1.06<br>[0.13; 8.33] | butorphanol+ dexmedetomidine | | | | | | | | | | |
| 0.27<br>[0.04; 2.12] | 2.90<br>[1.04; 8.06] | 0.43<br>[0.06; 3.24] | 0.57<br>[0.21; 1.53] | 0.67<br>[0.17; 2.67] | 0.70<br>[0.14; 3.62] | 1.08<br>[0.30; 3.84] | 0.98<br>[0.19; 5.00] | oxycodone | | | | | | | | | |
| 0.13<br>[0.01; 2.47] | 6.08<br>[0.15; 251.06] | 0.20<br>[0.00; 12.43] | 0.27<br>[0.01; 11.15] | 0.32<br>[0.01; 14.84] | 0.33<br>[0.01; 17.08] | 0.44<br>[0.01; 19.71] | 0.47<br>[0.01; 23.79] | 0.48<br>[0.01; 17.04] | remifentanil+ ketorolac | | | | | | | | |
| 0.13<br>[0.01; 2.39] | 6.30<br>[0.15; 259.98] | 5.08<br>[0.08; 309.41] | 0.26<br>[0.01; 10.76] | 3.22<br>[0.07; 148.98] | 3.11<br>[0.06; 159.07] | 2.34<br>[0.05; 104.18] | 0.45<br>[0.01; 22.97] | 2.17<br>[0.06; 77.69] | 1.04<br>[0.43; 2.52] | fentanyl +ketorolac | | | | | | | |
| 0.19<br>[0.04; 0.96] | 4.25<br>[0.87; 20.88] | 0.29<br>[0.03; 3.10] | 0.39<br>[0.08; 1.87] | 0.46<br>[0.07; 2.89] | 0.48<br>[0.06; 3.69] | 0.63<br>[0.11; 3.69] | 0.67<br>[0.09; 5.11] | 0.68<br>[0.20; 2.31] | 1.43<br>[0.05; 41.29] | 1.48<br>[0.05; 42.75] | sufentanil | | | | | | |
| 0.18<br>[0.01; 2.56] | 4.34<br>[0.61; 30.73] | 3.50<br>[1.11; 11.02] | 0.38<br>[0.06; 2.65] | 0.45<br>[0.05; 3.92] | 0.47<br>[0.04; 4.86] | 1.61<br>[0.55; 4.75] | 0.65<br>[0.06; 6.74] | 1.50<br>[0.28; 7.94] | 0.71<br>[0.01; 36.95] | 1.45<br>[0.03; 75.09] | 1.02<br>[0.13; 8.06] | morphine +ketorolac | | | | | |
| 0.08<br>[0.00; 1.82] | 9.55<br>[0.21; 443.11] | 0.13<br>[0.00; 8.80] | 0.17<br>[0.00; 7.98] | 0.20<br>[0.00; 10.58] | 0.21<br>[0.00; 12.15] | 0.28<br>[0.01; 14.07] | 0.30<br>[0.01; 16.93] | 0.30<br>[0.01; 12.26] | 1.58<br>[0.53; 4.71] | 0.66<br>[0.20; 2.16] | 2.25<br>[0.07; 73.75] | 0.45<br>[0.01; 26.28] | remifentanil | | | | |
| 7.42<br>[0.68; 80.79] | 5.92<br>[1.19; 29.47] | 4.77<br>[0.44; 51.17] | 2.50<br>[0.68; 9.16] | 3.03<br>[0.66; 13.96] | 2.92<br>[0.50; 17.15] | 2.20<br>[0.37; 12.96] | 2.08<br>[0.72; 6.00] | 3.24<br>[0.78; 13.37] | 0.97<br>[0.02; 42.83] | 0.94<br>[0.02; 41.36] | 1.39<br>[0.24; 7.91] | 1.36<br>[0.17; 10.88] | 0.62<br>[0.01; 30.60] | butorphanol | | | |
| 10.28<br>[0.58; 181.05] | 8.19<br>[0.86; 78.54] | 6.60<br>[0.38; 114.94] | 4.93<br>[0.68; 35.88] | 4.19<br>[0.46; 38.08] | 4.04<br>[0.37; 43.68] | 3.04<br>[0.28; 32.99] | 2.88<br>[0.43; 19.47] | 2.83<br>[0.38; 21.21] | 1.35<br>[0.02; 81.73] | 1.30<br>[0.02; 78.93] | 1.93<br>[0.18; 20.32] | 1.89<br>[0.14; 25.83] | 0.86<br>[0.01; 57.89] | 1.38<br>[0.28; 6.80] | buprenorphine | | |
| 0.03<br>[0.00; 0.54] | 29.92<br>[0.68; 1314.89] | 0.04<br>[0.00; 2.68] | 0.06<br>[0.00; 2.41] | 0.07<br>[0.00; 3.21] | 0.07<br>[0.00; 3.69] | 11.11<br>[0.23; 526.24] | 0.10<br>[0.00; 5.13] | 0.10<br>[0.00; 3.70] | 4.92<br>[1.60; 15.13] | 0.21<br>[0.11; 0.42] | 7.03<br>[0.23; 217.65] | 0.15<br>[0.00; 7.97] | 3.13<br>[0.80; 12.33] | 0.20<br>[0.00; 9.27] | 0.27<br>[0.00; 17.60] | oxycodone+ ketorolac | |
| 95.04<br>[1.27; 7103.07] | 75.79<br>[0.58; 9986.04] | 61.08<br>[0.34; 10898.99] | 45.64<br>[0.35; 5965.93] | 38.79<br>[0.27; 5573.79] | 37.36<br>[0.24; 5814.96] | 28.15<br>[0.20; 3930.75] | 0.04<br>[0.00; 5.81] | 26.14<br>[0.22; 3090.24] | 12.46<br>[0.51; 306.25] | 12.03<br>[0.47; 307.66] | 17.82<br>[0.18; 1798.28] | 17.45<br>[0.11; 2738.78] | 7.93<br>[0.39; 162.07] | 0.08<br>[0.00; 10.81] | 0.11<br>[0.00; 19.23] | 2.53<br>[0.09; 69.63] | dexmedetomidine |

Figure S3. Comparison-adjusted funnel plot assessing publication bias

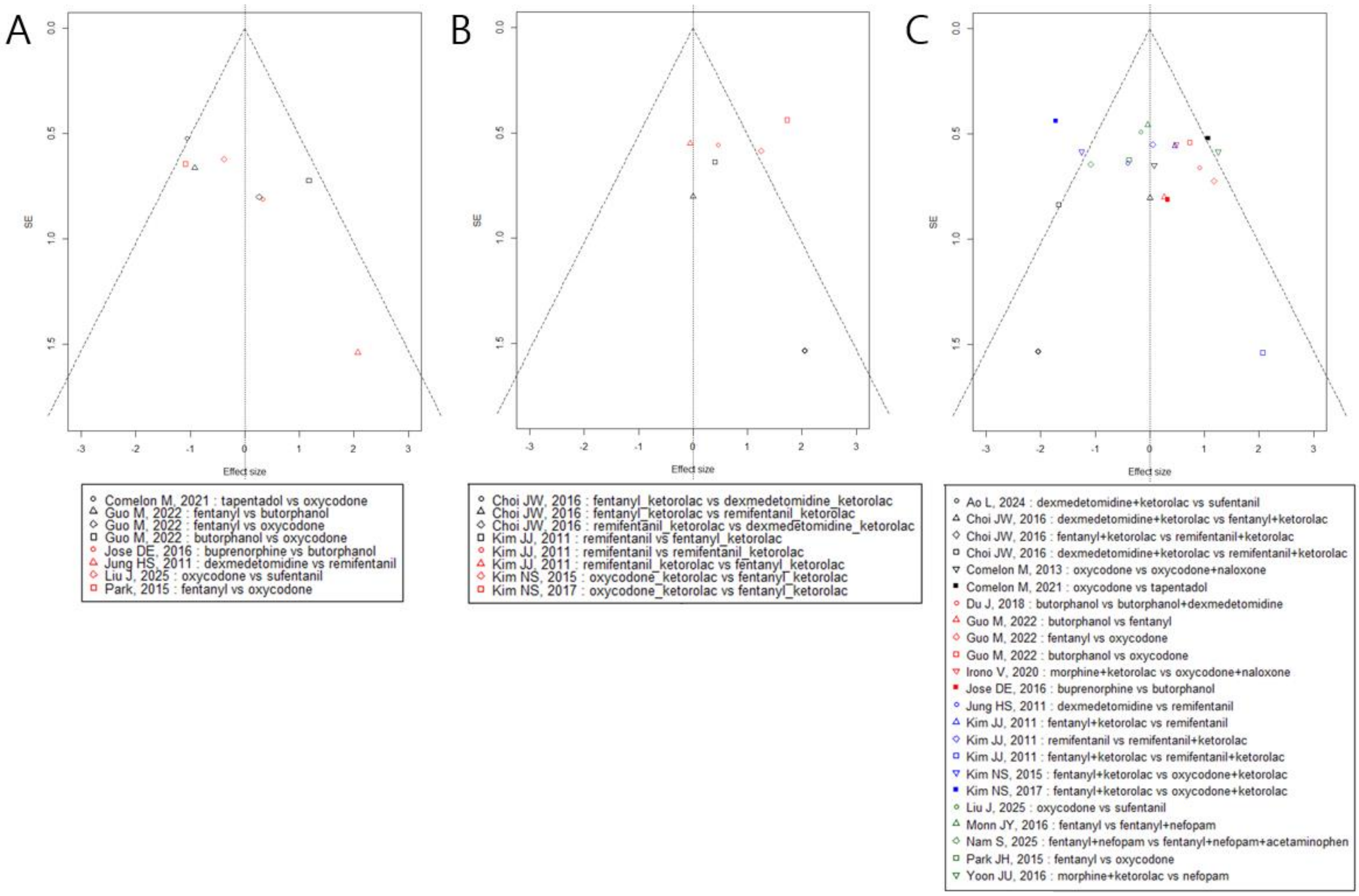


(A) Funnel plot of monotherapy subgroup.

(B) Funnel plot of combination therapies containing ketorolac.

(C) Overall funnel plot of all included comparisons.
